# Uptake and outcomes of a prevention-of mother-to-child transmission (PMTCT) program in Zomba district, Malawi

**DOI:** 10.1186/1471-2458-11-426

**Published:** 2011-06-03

**Authors:** Monique van Lettow, Richard Bedell, Megan Landes, Lucy Gawa, Stephanie Gatto, Isabell Mayuni, Adrienne K Chan, Lyson Tenthani, Erik Schouten

**Affiliations:** 1 Dignitas International, Zomba, Malawi; 2 Dalla Lana School of Public Health, University of Toronto, Toronto, Canada; 3 Department of Medicine at St Michaels Hospital, University of Toronto, Toronto, Canada; 4 Faculty of Health Sciences, Simon Fraser University, Burnaby, Canada; 5 Department of HIV and AIDS, Ministry of Health, Malawi; 6 Management Sciences for Health Lilongwe, Malawi

## Abstract

**Background:**

HIV prevalence among pregnant women in Malawi is 12.6%, and mother-to-child transmission is a major route of transmission. As PMTCT services have expanded in Malawi in recent years, we sought to determine uptake of services, HIV-relevant infant feeding practices and mother-child health outcomes.

**Methods:**

A matched-cohort study of HIV-infected and HIV-uninfected mothers and their infants at 18-20 months post-partum in Zomba District, Malawi. 360 HIV-infected and 360 HIV-uninfected mothers were identified through registers. 387 mother-child pairs were included in the study.

**Results:**

10% of HIV-infected mothers were on HAART before delivery, 27% by 18-20 months post-partum. sd-NVP was taken by 75% of HIV-infected mothers not on HAART, and given to 66% of infants. 18% of HIV-infected mothers followed all current recommended PMTCT options. HIV-infected mothers breastfed fewer months than HIV-uninfected mothers (12 vs.18, respectively; *p *< 0.01). 19% of exposed versus 5% of unexposed children had died by 18-20 months; *p *< 0.01. 28% of exposed children had been tested for HIV prior to the study, 76% were tested as part of the study and 11% were found HIV-positive. HIV-free survival by 18-20 months was 66% (95%CI 58-74). There were 11(6%) maternal deaths among HIV-infected mothers only.

**Conclusion:**

This study shows low PMTCT program efficiency and effectiveness under routine program conditions in Malawi. HIV-free infant survival may have been influenced by key factors, including underuse of HAART, underuse of sd-NVP, and suboptimal infant feeding practices. Maternal mortality among HIV-infected women demands attention; improved maternal survival is a means to improve infant survival.

## Background

Mother-to-child transmission (MTCT) of HIV accounts for 14% of all new HIV infections worldwide [1], and may occur during pregnancy, labor and delivery or breastfeeding. In the absence of prevention, rates of MTCT are estimated to be 25-35 percent [2,3]. In 2007, the national HIV prevalence rate among pregnant women in Malawi was 12.6% [4] with an estimated 89,000 HIV-infected children. Survival of these children is limited with approximately 50% dying before two years of age [2,4].

Since 2003, the primary prophylaxis regimen within the Malawian prevention of mother-to-child transmission (PMTCT) strategy has been single dose nevirapine (sd-NVP). At the time of study, PMTCT guidelines [2] in Malawi supported HIV testing and counseling (HTC) for all women presenting to antenatal clinics (ANC). HIV-infected women should have WHO clinical staging, CD4 count if indicated and available, and initiation of highly active antiretroviral treatment (HAART) for women in stages III or IV, or in stages I and II if CD4 count <250. All women not initiated on HAART should be provided sd-NVP during the antenatal period to be taken at the onset of labor and all HIV-exposed infants provided with sd-NVP syrup within 72 hours of birth [2]. Postnatal recommendations for HIV-exposed infants in Malawi were exclusive breastfeeding for the first 6 months of life, or if replacement feeding was acceptable, feasible, affordable, sustainable and safe, avoidance of all breastfeeding. Mothers were advised to return with the infant for HIV DNA-PCR testing at 6 weeks and counseling on follow-up HTC up to 18 months.

In 2003, the Ministry of Health (MoH) set a target of at least 75% of women attending ANC with PMTCT services by the end of 2010 [5]. By 2007, PMTCT services were available in 64% of health facilities [2]. In 2004, an evaluation of the PMTCT program in Thyolo district in Malawi revealed a progressive loss to follow-up of HIV-infected mothers; cumulative loss to follow-up was 55% by the 36 week antenatal visit, 68% by delivery and 81% by the 6 month post-natal visit [6]. In the same district, a qualitative study depicted many community and provider related operational and cultural barriers hindering the acceptability of the PMTCT program [7]. Although the provision of basic PMTCT services has been expanded considerably in Malawi in recent years, the efficiency and effectiveness of these interventions remain unknown. In Zomba district, where uptake of PMTCT services by pregnant women appears to be increasing, monitoring data showed that few HIV-infected mothers and HIV exposed infants are accessing HIV treatment, care and support and data on uptake and effectiveness of PMTCT interventions was limited. Assessing the quality of PMTCT services and identifying ways in which delivery of those services could be improved is essential to address maternal health needs and prevent MTCT.

## Methods

A matched-cohort study of HIV-infected and HIV-uninfected mothers and their infants 18-20 months after their estimated delivery date was conducted between August and December 2009.

This study was conducted in Zomba district (population 670,000) in southern Malawi where 80% of inhabitants are rural [8]. Antenatal surveillance data from sentinel health centers show that HIV prevalence is high, ranging from 12% to 34% [4]. Public health services are provided at one central hospital, one mission hospital and 33 health centers, run by the Zomba District Health Office (DHO). Clinical services and medications are provided by the MOH without user fees. Since 2005, the Zomba DHO in partnership with Dignitas International, have implemented PMTCT services. By 2007, 22 ANC sites in Zomba District provided PMTCT services. Zomba District data indicate that 96% of all women attend at least one ANC visit and that 56% of deliveries are performed by skilled birth attendants in health institutions [9].

20 out of 22 sites in the district where PMTCT services were provided for at least 18 months were included in this study. 2 sites, Zomba Central Hospital and St. Luke's Mission Hospital, were excluded as women who gave birth at these district level referral hospitals may not live in proximity and therefore would be more difficult to trace, and they may also be a different population than those who attend health centres. All HIV-infected mothers who attended one of the 20 rural public facilities that provide antenatal services, and had an estimated delivery date between March 1st and May 31st 2008, were identified through antenatal, delivery and postnatal facility registers. By reviewing all available registers, we attempted to capture the entire cohort of HIV-infected pregnant women in the district. For every HIV-infected mother, the next registered HIV-uninfected mother was identified as a control. 5 other health centers were excluded during this process since few (<10) HIV positive women were identified.

Community sensitization meetings were conducted prior to data collection to inform community leaders and health facility teams about the study. In addition, study advertisement posters were displayed at health centers and in the villages. Health Surveillance Assistants (HSAs) helped trace participants and asked the identified mother-child pairs to come to the health centre for interviews. Since equal numbers of HIV-infected and HIV-uninfected mothers were recruited there was no association between study participation and HIV status. In cases where the mother had died, the child's primary caregiver was interviewed. If the mother or the primary caregiver did not come for interview, the interviewer, accompanied by the HSA, visited the participant at home for the interview. In cases where study participants were reported to have moved or died, village headmen were consulted and village registers reviewed for verification.

Data collection was conducted by 6 trained female interviewers through semi-structured interviews using a standardized questionnaire.The questionnaire administered to the mothers included questions regarding socio-economic demographics, parity, disclosure of HIV status, partner testing, uptake of PMTCT services from HTC at ANC to uptake of sd-NVP and HAART, and feeding options utilized. Information concerning prior HIV-testing and ARVs was verified through personal health passports, a small personal paper booklet that provides a record of clinical encounters. Responses on uptake of sd-NVP or HAART were verified with available information in health passports and registers. When information differed or was missing (e.g. when mother had died) the information from the registers was included.

Mothers and/or children with negative or unknown HIV status were offered point of care HIV rapid testing. All testing was done by trained counselors as per the Malawian MOH guidelines. Mothers and/or children found HIV-infected were referred to the nearest clinic which could provide appropriate ART services, including ongoing care and assessment for ART eligibility.

Data and statistical analysis were conducted using STATA 9.1 (StataCorp, Texas, USA) and SPSS 17.0 (SPSS, Inc., Chicago, IL, USA). Comparisons between groups were made using *t*-tests and exact tests. Relative risks (RR) as a ratio of two proportions with a confidence level of 95% were derived from two-by-two contingency tables. Reported RR's are unadjusted, unless stated otherwise and all variables were considered significant with a *p *< 0.05. HIV-free survival by 18-20 months was calculated as: total number of cohort minus number of transmissions, deaths and not tested by 18-20 months divided by total number of cohort minus number not tested.

This study received ethical approval from the National Health Science Research Committee in Malawi. Written informed consent was given by all participants.

## Results

### Demographics of Mother-Child Pairs

Table 1 provides an overview of mother-child pairs identified and traced. 360 HIV-infected mothers and 360 HIV-uninfected mothers were identified through all available registers. 173 HIV-infected and 214 HIV-uninfected mothers-child pairs were found and included in the study. Out of the 720 mother-child pairs traced, 61 had moved out of the area, 265 were not found and 3 were not willing to participate. No significant difference was found in the proportion of mothers having moved out of the area or not found (*p *= 0.09). 6 caregivers responded for a deceased mother and 40 mothers with children that had died were included alone. 5 mother-child pairs were confirmed to have both died.

**Table 1:** Cohort Description

	HIV-infected Mothers	HIV-uninfected Mothers
Total number of mother-child pairs traced	360	360
Moved out of the catchment area	37 (10%)	24 (7%)
Not found	144 (40%)	121 (34%)
Not willing to participate	3 (1%)	0 (0%)
**Total mother-child pairs included**	**173 (48%)**	**214 (59%)**
Both mother/child died	5	0
Child only died	29	11
Mother only died	6	0

In this cohort, HIV-infected mothers were older than HIV-negative mothers; median 29 vs. 24 years of age, respectively; *p *= 0.01. HIV-infected mothers were less likely to have received secondary education than HIV-uninfected mothers (RR 0.5; 95%CI 0.3-0.8; *p *= 0.001). No significant differences were found between HIV-infected and HIV-uninfected mothers in terms of employment status, dwelling type, water source or transport method used, indicating a uniformly poor community.

14 (9%) HIV-infected mothers identified by registers denied or did not want to reveal their HIV status. HIV-infected mothers were four times more likely not to have disclosed their HIV status to their partners than HIV-uninfected mothers (RR 3.5; CI 95% 1.3-9.7; *p *= 0.008) and partners of HIV-infected mothers were less likely to have gone for HIV testing than partners of HIV-uninfected mothers (RR 0.7; 95%CI 0.6-0.9; *p *< 0.001).

Median parity among HIV-infected and HIV-uninfected mothers was 4 (IQR1-7) and 2 (IQR 0-5), respectively; *p *= 0.001. When controlling for age HIV-infected women were still more likely to have more than 2 children than HIV-uninfected women. (age adjusted RR 2.3; CI 95% 1.3-4.5; *p *= 0.007).

### Uptake of PMTCT services

Tables 2 and 3 give an overview of uptake of PMTCT services, from HTC at ANC to uptake of sd-NVP and HAART and feeding options utilized. Mothers reported to have attended a mean and median of 4 ANC visits (IQR 1-5) with no difference between HIV-infected and HIV-uninfected mothers (*p *= 0.54). 97% of all mothers were offered HTC and 96% underwent HTC during ANC visits.

**Table 2:** Uptake of ANC, HTC and recommended feeding options

	HIV-infected (n = 173)	HIV-uninfected (n = 214)	*p*-value
***HTC at ANC and labor***			
**Mean number of ANC visits **(±SD)	3.7 (±1.3)	3.8 (±1.2)	0.54
**Offered HTC at ANC**	158/162* (97.5%)	207 (96.7%)	0.12
**Uptake of HTC at ANC**	154/162 (95.1%)	206 (96.3%)	0.22
**Known HIV status before labor**	146/162 (90.1%)	195 (91.1%)	0.23
**Received HTC at or within 48 hrs after labor (If status not already known)**	7/19 (36.8%)	5/16 (31.3%)	0.55
*** Utilization of feeding options***			
**Avoidance of all breastfeeding from birth**	3 (1.8%)	2 (0.9%)	
**6 months exclusive breastfeeding and NO breastfeeding after**	34 (19.7%)	2 (0.9%)	0.002
Mixed feeding** started within the first 6 months	58 (33.5%)	73 (34.3%)	
6 months exclusive breastfeeding and mixed feeding* from 6 months onwards.	75 (43.4%)	136 (63.8%)	
**Mean number months breastfed (±SD)**	12.1 (± 6.4)	17.9 (± 3.4)	<0.001

*162 alive HIV-infected mothers

**Mixed feeding: breast, commercial infant formula and other

**Table 3:** Uptake of antiretrovirals among HIV-infected mother-child pairs (N = 173)

*Uptake of maternal and infant sd-NVP during pregnancy and labor*	
**Maternal sd-NVP received at ANC **by those not already on HAART	111 (67.7%)
**Maternal sd-NVP taken at onset or later in labor by mothers not on HAART **(n = 156)	117 (75.0%)
Infant sd-NVP received within 72 hours after birth	114 (65.9%)
***Uptake of maternal ART***	
**Mothers already on HAART at ANC**	9 (5.2%)
**Mothers started HAART during pregnancy**	8 (4.6%)
**Mothers that started HAART post-partum**	28 (23.9%)
**Mothers attended (pre-) ART follow up in past 6 months**	72 (44.4%)
**ART**	45 (100%)
**pre-ART**	27 (23.1%)

*** Uptake of follow up for exposed infants***	
**Alive Mothers advised about follow up for exposed infants**	99 (61.1%)
**Infants followed and tested for HIV at least once at 18-20 months of age**	47 (27.6%)
**Exposed children tested HIV-Infected prior to the study**	7/47 (14.9%)
**HIV+ children started HAART**	2

Of the HIV-infected mothers, 9 (5%) were already on HAART before attending the ANC clinic. 100 (65%) of the mothers not already on HAART at ANC reported they were referred and attended (pre-) ART services at least once, and 8 (5%) mothers initiated HAART during pregnancy. Out of the 17 mothers on HAART during pregnancy and delivery 15 (94%) of their infants received sd-NVP immediately after or within 72 hours after birth, and 8 (50%) of the mothers on HAART followed a recommended feeding strategy.

In addition, 111 of the 164 other HIV-infected mothers (68%) that were not yet on HAART were provided with sd-NVP and were instructed to take it at the onset of labor. 75% of mothers not on HAART were reported to have taken the maternal sd-NVP at onset of or received their dose during labor. 66% of the exposed infants were reported to have received sd-NVP immediately or within 72 hours after birth.. Only 22 (14%) of the mothers not on ART followed all recommended strategies meaning both mother and infant took sd-NVP and the recommended feeding option was utilized. Overall among HIV-infected mothers, 30 (18%) were able to follow all current PMTCT recommendations.

341 of all women (91%) reported to that they knew their HIV status before going into labor. Among the 35 women with unknown HIV status, 12 (34%) were tested during or within 48 hours after labor.

45 (26%) HIV-infected mothers were on HAART by 18-20 months post-partum (at the time of the study) and reported having an ART follow up visit in the last 6 months. 27 (23%) HIV-infected mothers not on HAART reported having a pre-ART follow-up visit in the past 6 months. Of the 128 HIV-infected mothers not on ART, 56 (44%) reported not having started based on high CD4 (WHO stage I or II). The remaining 72 HIV-infected mothers (56%) were either never staged or had an unknown CD4.

Among the HIV-infected mothers, 99 (61%) recalled being told about the importance of follow up for their exposed infants. Of all exposed infants, 47 (28%) attended follow up for HIV-testing at least once.

HIV-infected women were more likely to opt for 6 months exclusive breastfeeding compared to HIV-uninfected women; 20% versus 1% respectively (*p *= 0.002). HIV-infected mothers breastfed fewer months than HIV-uninfected mothers (mean 18 vs. 12 months, respectively; *p *< 0.01), and 48% of HIV-infected and 90% of HIV-uninfected mothers were still breastfeeding at >18 months (*p *< 0.01). 28 HIV-infected women started HAART post-partum at a mean of 9.5 months into the breastfeeding period.

Table 4 shows the proportion of recommended PMTCT strategies followed by the cohort of HIV-infected mothers and their infants.

**Table 4:** Summary of uptake of recommended PMTCT strategies amongst HIV-infected mothers (N = 173)

		sd-NVP at the onset of labor	Infants given sd-NVP*	Recommended BF option followed**	followed all*** current PMTCT recommendations
Mothers on HAART	17		15/16 (94%)	8/16 (50%)	8/15 (53%)
Mothers NOT on HAART	156	117/156 (75%)	99/154 (64%)	29/154 (19%)	22/154 (14%)
Total	173	117/156 (75%)	114/170 (67%)	37/170 (22%)	30/170 (18%)

*Live-born infants only included

**Avoidance of all breastfeeding from birth or exclusive breastfeeding for the first 6 months of life, and no breastfeeding after.

***Mothers on HAART during pregnancy and delivery AND infants received sd-NVP AND recommended feeding options were utilized; OR mothers had taken sd-NVP at onset or during labor AND infants had received sd-NVP AND recommended feeding options were utilized.

### Maternal and Child Mortality and HIV Transmission

Table 5 shows mother and child outcomes by HIV status. There were 11 (6.4%) maternal deaths, all among HIV-infected women. The mean age of the child at time of mother's death was 10 months. Among the 5 mother-child pairs where both died, 3 children died within 3 months after their mothers' death; 2 children died within 6 months prior to the death of their mothers. Children of HIV-infected mothers were four times more likely to have died by the time of study than those of HIV-uninfected mothers (19% vs. 5%; RR3.9; 95%CI 2.1-7.5; *p *< 0.001).

**Table 5:** Maternal and Child Mortality and HIV Transmission

	HIV-infected N = 173	HIV-uninfected N = 214	*p*-value
**Maternal Deaths**	11 (6.4%)	0	
**Mean Child's Age at Maternal Death in months **(±SD)	10.3 (±4.8)	0	.
**Child Deaths**	34 (19.1%)	11 (5.1%)	0.0001
**Child Death Type**			
Illness	30 (17.3%)	9 (4.2%)	0.001
			.
Accident	1 (0.6%)	0	.
Stillbirth < 36 weeks	1 (0.6%)	1 (0.5%)	0.38
Stillbirth at term	1 (0.6%)	1 (0.5%)	
Dead within 48 hours	1 (0.6%)	0	
**Mean Age at child death in months **(±SD)	9.2 (±6.2)	7.1 (±4.9)	
**HIV Testing at the time of study**			
	**Alive children born from HIV-infected mothers (n = 139)**	**Alive children born from HIV-uninfected mothers (n = 203)**	
**Number of children tested at time of study**	104 (74.8%)	148 (72.9%)	0.59
**Total HIV Transmissions**			
Known prior to study	7	0	
Tested in study (new diagnosis)	8	0	
**Total**	**15**	**0**	
(**HIV-free**) **survival by 18-20 months **(95% CI)	66.2% (58.4-74.0)	93.1% (89.1-97.9)	0.0001

**Figure 1:**
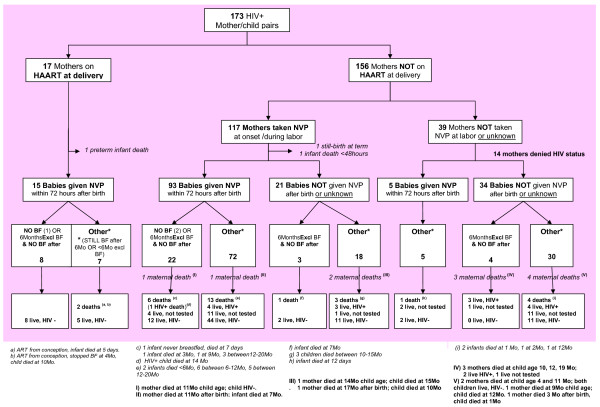
**PMTCT uptake, infant feeding practices and mother-child outcomes**.

Figure 1 gives a detailed overview of the uptake of PMTCT services and infant feeding practices utilized by HIV-infected mothers and their infants. As part of the decision chart mother and child outcomes, in terms of maternal and child mortality and HIV transmission related to the uptake of the PMTCT recommendations, are shown by strategy followed.

HTC was offered to all HIV-uninfected mothers, 154 (72%) were tested and 4 (2.6%) new sero-conversions were confirmed at the time of study. As 40% of the uninfected mothers were lost to follow, this percentage may be an underestimation. HTC was also offered to the children, of which 104 (75%) exposed children with unknown status were tested and 8 new diagnoses were confirmed. Given that 7 children were known HIV-infected prior to the study, the total number of transmissions among living tested children was 15 (13.5%). HIV-free infant survival at 18-20 months among HIV-exposed was 66.2% whereas survival among HIV-unexposed infants was 93.1%.

## Discussion

This study assesses PMTCT program outcomes under routine conditions in Zomba District and identifies areas for improvement. Overall PMTCT effectiveness indicated by HIV-free survival at 18-20 months was much lower under routine program conditions than results from study populations would suggest. Data from the HIVNET012 trial in Uganda showed that in the study arm that received maternal and infant sd-NVP HIV free survival was 79.7% (95% CI 74.7-83.8) by 18 months. Our results show even lower HIV-free survival of 66% (95% CI 58.4-74.0).

We found suboptimal use of NVP prophylaxis with 75% of mothers not on HAART taking sd-NVP at onset or during labor and 66% of infants receiving sd-NVP within 72 hours after birth. Similar poor uptake was shown in Uganda, where 77% of HIV-infected pregnant women received therapy, and 62% of the reported exposed infants received newborn sd-NVP [10]. Poor uptake may in part be related to an unwillingness of mothers to reveal their HIV status, since we know that the 14 (9%) HIV-infected mothers who denied their status in this study were among the least likely to comply with the recommended strategies. In concurrence, recent qualitative studies from Malawi found that low uptake of or dropping out of PMTCT services was related to avoidance of involuntary HIV disclosure, fear of negative community reactions and lack of support from husbands [11,12].

The results of this study suggest there was under-prescription of HAART for pregnant women. Only 10% of HIV-infected mothers were on HAART during pregnancy and delivery and half of those initiated HAART during the reference pregnancy for this study. This low percentage of women on HAART may be related to a lack of clarity of recommendations or because staging and CD4 counts were underperformed. Over half of the HIV-infected pregnant women were either never staged or had an unknown CD4 count. While this may have contributed to potentially preventable MTCT, it is also likely a major factor related to maternal death. Maternal mortality among HIV-infected women was very high at 6% (equivalent to 6000 per 100000) whereas there were no deaths among HIV-uninfected mothers.

Recommended infant feeding practices (current at that time) were poorly adhered to as HIV-infected mothers not on HAART breastfed on average 12 months and 48% were still breastfeeding after 18 months. This may also be related to lack of clarity of infant feeding recommendations, to stigma associated with not breastfeeding, and/or to lack of safe alternatives to breast milk. Other recent studies from Malawi, Kenya and Zambia described this lack of clarity among health workers and the low priority for infant feeding counseling within PMTCT programs [11-13]. Taha and colleagues conducted a series of trials in Malawi studying the effectiveness of NVP and combination therapy and the impact of breastfeeding on MTCT [14-17]. For infants not infected at birth and retested at 6 to 8 weeks, transmission was 6.5% [15,16]. By the age of 24 months, the cumulative risk of HIV infection among infants who received short antiretroviral prophylactic regimens and were uninfected at 1.5 months was 9.7% [17] This suggests that even with the proper application of sd-NVP there was likely to be significant continued vertical HIV transmission throughout the breast feeding period among our study population (unless the mother was taking HAART).

Follow-up HIV testing for HIV-exposed infants was poor. Only 28% of exposed infants were followed and tested at least once by 18-20 months of age (prior to this study). Recent studies from Mozambique, Kenya and Uganda show similar low uptake and high drop out rates of HIV-exposed infants and children for HIV diagnoses and care [18-21]. Loss to follow up of exposed infants and children among our study population reflects a lack of systematic follow-up by health facilities as well as some lack of knowledge or denial of the importance of infant follow-up among HIV-infected mothers.

The most striking finding of this study is that among all HIV-infected mothers, only 18% were able to follow all current PMTCT recommendations. Other studies have shown similar low coverage of PMTCT services in sub-Saharan Africa [22-24]. Partner and community involvement have been described as strategies for improving this low coverage [10-12]. New and innovative research is needed to inform programmers about barriers and facilitators to uptake of PMTCT services and safe infant feeding options. Improved HIV-free infant survival, and improved maternal survival, may be achieved by optimizing initiation of HAART during pregnancy and breastfeeding [25,26]. Improved post-natal follow-up could better support mothers in adhering to safe infant feeding options to minimize post-natal vertical HIV transmission and also allow for timely diagnosis of infants who acquire HIV infection despite PMTCT interventions, or when none were implemented.

In 2011, the Malawi MoH is planning a test and treat strategy to optimize delivery of HAART during pregnancy and breastfeeding as recommended in the revised WHO guidelines (Option B plus lifelong HAART), and the results of this study provide strong local evidence to support these plans. The low compliance we observed with a relatively simple PMTCT intervention such as sd-NVP suggests that uptake of maternal HAART during pregnancy and the breastfeeding period is likely to face similar or potentially greater challenges (due to the regimen's greater complexity) in its programmatic application. Concurring, Rollins and colleagues who studied the effect of health systems performance on rates of MTCT concluded recently that the introduction of more effective combination ART interventions will yield only marginal reductions in childhood HIV infections and mortality unless health systems achieve high levels of performance at each step of the PMTCT pathway [27].

## Conclusion

This study shows low PMTCT program efficiency and effectiveness under routine program conditions in Malawi. PMTCT effectiveness indicated by HIV-free survival at 18-20 months was much lower under routine program conditions than results from study populations would suggest.

Maternal mortality among HIV-infected women was high and demands further attention as improved maternal survival is also a means to improve infant survival. Improved HIV-free infant survival, and improved maternal survival, may be achieved by optimizing initiation of HAART during pregnancy and breastfeeding. Further operational research is needed to examine the institutional and maternal factors that influence the uptake of PMTCT services and maternal ART. Failures at various points in the PMTCT cascade may be associated with avoidable MTCT

### Limitations of the study

This study was limited to the cohort of women that were identified through health centre registers, and therefore had had at least one potential interaction with formal PMTCT services. Our exclusion criteria excluded women registered only at tertiary referral centres and it is possible that these women had different characteristics than our study population (e.g. urban, wealthier, high risk obstetrical problems). Some information could not be verified (e.g. infant feeding options utilized) and was based on maternal recall only. Finally, when tracing women to their homes, interviewers were not always able to be accompanied by a HTC counselor for testing resulting in a lower proportion of women and infants tested. Mothers and children were subsequently referred to the health centre for HTC, but these results were not available for inclusion in the study.

## Competing interests

The authors declare that they have no competing interests.

## Authors' contributions

Conceived and designed the study: MvL, RB, ML, SG; Conducted the study and acquisition of data: SG, LG, IM; Analysis and interpretation of data: MvL, RB, ML; Wrote the manuscript: MvL, RB, ML; Critical review of manuscript: AKC, LT, ES. All authors read and approved the final manuscript.

## Pre-publication history

The pre-publication history for this paper can be accessed here:

http://www.biomedcentral.com/1471-2458/11/426/prepub
